# DEM Study and Field Experiments on Coupling Bionic Subsoilers

**DOI:** 10.3390/biomimetics10050306

**Published:** 2025-05-11

**Authors:** Zihe Xu, Hongyan Qi, Lidong Wang, Shuo Wang, Xuanting Liu, Yunhai Ma

**Affiliations:** 1 The College of Biological and Agricultural Engineering, Jilin University, 5988 Renmin Street, Changchun 130025, China; zhxu21@mails.jlu.edu.cn (Z.X.); wangshuo21@mails.jlu.edu.cn (S.W.); xuantingl20@mails.jlu.edu.cn (X.L.); 2 The Key Laboratory of Bionic Engineering, Ministry of Education, Jilin University, 5988 Renmin Street, Changchun 130025, China; 3 College of Mechanical Engineering, Hunan University of Arts and Science, Changde 415000, China; qhy@huas.edu.cn; 4 Weichai Power Co., Ltd., Weifang 261061, China; wanglidong@weichai.com

**Keywords:** bionic design, subsoiler, discrete element method (DEM), draft force reduction

## Abstract

Subsoiling is an effective tillage method for breaking up the plough pan and reducing soil bulk density. However, subsoilers often encounter challenges such as high draft resistance and excessive energy consumption during operation. In this study, the claw toes of the badger and the scales of the pangolin were selected as bionic prototypes, based on which coupling bionic subsoilers were designed. The discrete element method (DEM) was used to simulate and analyze the interactions between soil and both the standard subsoiler and coupling bionic subsoilers. Field experiments were conducted to validate the simulation results. The simulation results showed that the coupling bionic subsoilers reduced the draft force by 7.70–16.02% compared to the standard subsoiler at different working speeds. Additionally, the soil disturbance coefficient of the coupling bionic subsoilers decreased by 5.91–13.57%, and the soil bulkiness was reduced by 2.84–18.41%. The field experiment results showed that coupling bionic subsoilers reduced the average draft force by 11.06% and decreased the soil disturbance area. The field experiments validated the accuracy of DEM simulation results. This study provides valuable insights for designing more efficient subsoilers.

## 1. Introduction

Subsoiling is an important part of conservation tillage. It can alleviate soil compaction, improve the soil water-holding capacity, and has been widely used [1,2]. Additionally, subsoiling can improve crop growth conditions, increase root depth, and crop yield [3]. The subsoiler, as the primary tool for subsoiling operation, breaks the plough pan with minimal disturbance to the topsoil and surface crop residues. However, its deep working depth and the adhesive nature of soil contribute to high tillage resistance and energy consumption, limiting the economic benefits and popularization of conservation tillage [4,5]. Therefore, reducing the draft force of subsoilers is essential for improving operational efficiency and sustainability.

The subsoiler is a type of soil-engaging component. Currently, methods for reducing the resistance of soil-engaging components primarily include bionic design, vibration, electro-osmosis, and surface modification [6,7,8,9]. Among these, bionic design, as an innovative approach inspired by the structure and function of creatures in nature, has gained considerable research attention [10]. Researchers have observed and analyzed biological characteristics, particularly those of soil-dwelling animals. These insights are then applied to improve soil-engaging components’ performance.

Zhang et al. [11] designed a subsoiler by integrating the inner and outer contour curves of mole cricket claws. Compared with the common subsoiler, the bionic subsoiler reduced the draft force by 16.34% in the horizontal direction and by 24.53% in the vertical direction. Song et al. [12] extracted the structural characteristics of mole claws and applied them to a standard subsoiler, demonstrating that optimizing the subsoiler geometry through bionic design can effectively reduce the draft force. In addition, researchers have identified that non-smooth surface structures observed in organisms such as earthworms, dung beetles, and pangolins can significantly reduce resistance. Wu et al. [13] designed a subsoiler tine with a bionic non-smooth surface structure, achieving a reduction in draft force of 24.6–33.7% compared to a smooth subsoiler tine. Wang et al. [14] applied the shark scale structures to the surfaces of the subsoiler shank and tine, demonstrating a resistance reduction advantage during field operations. These bionic structures were able to reduce the soil-contact area under suitable working conditions, thereby decreasing soil adhesion and resistance.

Despite these advancements, most previous designs of bionic subsoilers have relied on a single bionic prototype. However, soil–tool interactions involve multiple processes, including cutting, friction, and adhesion. A single bionic design strategy may limit the potential synergistic effects of combining multiple natural drag-reduction mechanisms, thereby constraining further performance optimization. Coupling bionic design provides a new approach to overcoming these limitations and is playing a more and more significant role in the bionic implement of biological function [15]. Gao et al. [16], based on the theory of biological coupling, developed a bionic drill bit inspired by multiple creatures. This bionic drill bit reduced resistance and adhesion during rock cutting, resulting in a higher drilling speed. Wang et al. [17] combined the structural characteristics of pangolins and dung beetles to design a coupled bionic furrow opener, which demonstrated a maximum draft reduction of 13.21%. Liu et al. [18] developed coupling bionic excavator buckets modeled on the curves of the earthworm and pangolin claw toe. A maximum drag reduction of 14.47% was achieved compared to the prototype bucket. These studies demonstrate that the synergistic optimization of multiple biological features can overcome the performance bottlenecks of single-bionic designs.

Currently, few studies have attempted to couple more than one biological inspiration in subsoilers. Therefore, inspired by the structural characteristics of badger claws and pangolin scales, guided by the functionality principle of coupling bionic design, this study developed a coupling bionic subsoiler with low-resistance characteristics. The curvature of the badger claw was incorporated into the design of the subsoiler blade to achieve efficient soil cutting and lifting, while the grooved structure of the pangolin scale was applied to the sidewalls of the subsoiler to further reduce draft force and enhance tillage efficiency.

The structure and operating parameters of the subsoiler also significantly influence resistance and soil disturbance. To gain comprehensive insights into the performance of the coupling bionic subsoiler, it is essential to accurately evaluate its interactions with soil. To date, the discrete element method (DEM) has been widely applied to simulate soil–tool interactions, including studies on cutting blades [19,20], digging shovels [21,22], harvesting tools [23,24], and subsoilers [11,25]. The above research indicates that DEM plays a crucial role in evaluating tillage resistance and soil movement.

In this study, coupling bionic subsoilers were designed based on the structural characteristics of badger claws and pangolin scales. The discrete element method was utilized to analyze the forces acting on the subsoiler and the soil disturbance effects under different tillage speeds. Field experiments were conducted to evaluate and compare the performance of the bionic subsoiler with that of a conventional standard subsoiler, thereby validating the effectiveness and optimization potential of the coupling bionic design. This research offers valuable insights for the optimization and enhancement of subsoilers and other soil-engaging components.

## 2. Materials and Methods

### 2.1. Design of Coupling Bionic Subsoiler

#### 2.1.1. Bionic Structure Extraction

The badger has exceptional digging ability. Among its five toes, the middle toe is the longest and the first to contact the soil during excavation, playing a key role in soil breaking [26]. In this study, the middle toe of the front claw was selected as the research object. After cleaning and disinfecting the claws, a stereomicroscope (Olympus SZX12) was used to capture images of its longitudinal cross-section. Image processing techniques were then applied to fit a curve to the extracted contours. The images were imported into MATLAB 2021, where the inner contour curve of the claw toe was extracted. The following equation is the claw fitting curve equation.

(1) $$y=2.542{\times 10}^{-8}x^{4}-2.062{\times 10}^{-5}\;x^{3}+0.006793\;x^{2}-0.3758x+27.1$$

where x and y are the pixel coordinates of the claw’s inner contour. The correct definition of R^2^ is 0.99, which means this mathematical model can accurately represent the characteristics of the claw.

**Figure 1:**
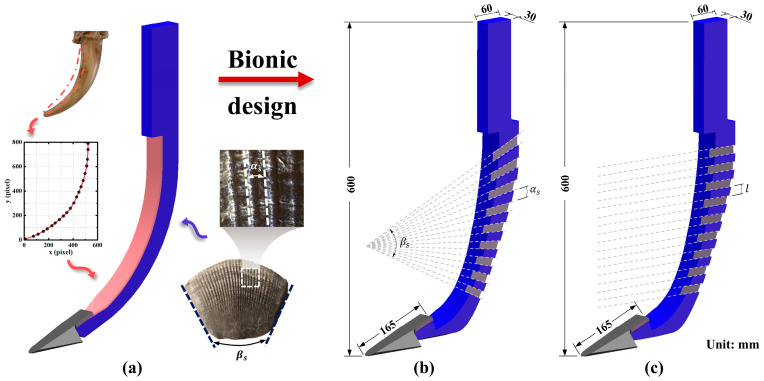
Design of coupling bionic subsoiler. (**a**) Conventional standard subsoiler (CSS). (**b**) Coupling bionic subsoiler with angled grooves (CBSA). (**c**) Coupling bionic subsoiler with horizontal grooves (CBSH).

The pangolin scale was selected as another bionic prototype. The surface of pangolin scales features ribbed structures, which exhibit low resistance and reduced soil adhesion [27,28]. Before analysis, the scales were cleaned and dried. A stereomicroscope was used to capture images of the surface structures. Longitudinal ridges were observed to be arranged along the surface of the scales, with an included angle $\alpha_{s}$ between adjacent ridges, as indicated by the white dashed lines in Figure 1a. Additionally, an expansion angle $\beta_{s}$ was formed along the lateral edges of the scales, as shown by the black dashed lines in Figure 1a. The captured images were imported into CAD software for quantitative analysis, where these angular parameters were measured. The results showed that the average included angle $\alpha_{s}$ between adjacent longitudinal ridges was 3°, and the average expansion angle $\beta_{s}$ of the scale was 51°. These structural parameters and the curve equations were subsequently applied to the design of the coupling bionic subsoiler.

#### 2.1.2. Three-Dimensional Modeling of Coupling Bionic Subsoiler

In this study, a conventional standard subsoiler (CSS) compliant with the Chinese standard (JB/T 9788-2020) was used as the basis. The subsoiler consists of a sweep tine and a curved shank. The sweep tine increases the soil disturbance range, while the curved shank reduces draft resistance during tillage [29]. Nearly half of the resistance encountered by the subsoiler originates from the shank, as the cutting edge cuts and breaks up the soil, while the lateral surfaces of the shank experience friction and adhesion from the soil [30]. To address this, the concept of coupling bionics was employed to design a low-resistance subsoiler, integrating the characteristics of two biological prototypes into the subsoiler shank.

The extracted badger claw contour was scaled appropriately and applied as the cutting edge curve of the bionic subsoiler. Compared to the conventional standard subsoiler edges, the bionic subsoiler edge exhibits greater curvature variation. However, directly modifying the standard subsoiler edge into a bionic curve would result in an excessively narrow shank end, potentially leading to stress concentration and fracture during operation. To prevent this, the rear contour of the subsoiler shank was adjusted while maintaining the same side surface area as the standard shank. The groove structure inspired by pangolin scales was applied to the lateral surface of the subsoiler shank. The groove arrangement significantly affects performance. If the grooves are spaced too far apart, more soil will contact the surface, impeding soil flow. Conversely, excessively dense spacing creates a more continuous interface, which is less effective in reducing resistance [31].

Two types of coupling bionic subsoilers were designed. The first type, the coupling bionic subsoiler with angled grooves (CBSA), is shown in Figure 1b. In CBSA, the bionic structures on the lateral surfaces of the subsoiler shank were arranged at an interval of angle $\alpha_{s}$ ($\alpha_{s}$ = 3°), which corresponds to the angle formed by adjacent grooves on pangolin scales. The second type, the coupling bionic subsoiler with horizontal grooves (CBSH), is illustrated in Figure 1c. In CBSH, the bionic structures on the lateral surfaces of the subsoiler shank were distributed horizontally along the working direction of the subsoiler, with an interval of l (l = 15 mm). The bionic structures of the side walls of the two types of bionic subsoilers are located at the same height. Detailed dimensions are provided in Figure 1b,c. Three-dimensional models of the subsoilers were created in SolidWorks 2018 and manufactured using 65Mn steel. These subsoilers were evaluated through DEM simulations and field experiments, respectively, to analyze the effectiveness in reducing resistance and improving soil disturbance.

### 2.2. Discrete Element Simulation Modeling

#### 2.2.1. Simulation Parameters and Soil Modeling

DEM is an effective numerical approach for studying the dynamic behavior of granular media and serves as a useful tool for optimizing the design of soil-engaging components [32]. In this study, EDEM 2020 software was used to simulate the subsoiling process. The accurate setting of parameters is crucial for ensuring simulation reliability. The DEM simulation parameters primarily include material parameters and contact parameters.

Material parameters for the soil model were obtained through field sampling conducted in Gongzhuling City, Jilin Province (124.59° E, 43.39° N), in northeastern China. Due to the tillage characteristics of this region, a plough pan is commonly present, and the soil can be commonly divided into three layers: the plough layer, the plough pan, and the subsoil layer. The measured densities for these layers were 2580, 2630, and 2610 kg/m^3^, respectively, with bulk densities of 1290, 1430, and 1370 kg/m^3^. The particle diameter of the soil significantly influences both computational accuracy and simulation time. Smaller particle diameters can obtain more accurate results but drastically increase computational costs. Previous studies found that using soil particles with a 5 mm radius can accurately predict draft and vertical forces under various soil conditions [33]. Therefore, in this study, the nominal radius of soil particles was set to 4 mm, with the particle size randomly generated within a range from 95 to 105% of the nominal size.

The material of the subsoiler in the simulation was set as 65Mn steel, consistent with the actual subsoiler used in field experiments. The Hertz–Mindlin with Johnson–Kendall–Roberts (JKR) interaction model was selected to define the contact behavior between particles. This model accounts for the cohesive forces between wet soil particles by characterizing their mutual attraction through surface energy. Combining elastic deformation with van der Waals forces within the contact area, this model effectively simulates particle adhesion caused by moisture, making it more suitable for simulating cohesive soil [34]. The governing equation for this model is as follows:

(2) $$F_{JKR}=-4\sqrt{\pi \gamma E^{*}}\alpha^{\frac{3}{2}}+\frac{4E^{*}}{3E^{*}}\alpha^{3}$$

(3) $$\delta =\frac{\alpha^{2}}{R^{*}}-\sqrt{\frac{4\pi \gamma \alpha }{E^{*}}}$$

where $F_{JKR}$ is the normal contact force in the JKR contact model, γ is the surface energy, $E^{*}$ is the equivalent elastic modulus, α is the contact radius between the two particles, δ is the normal overlap of two particles, and $R^{*}$ is the equivalent radius.

The equivalent Young’s modulus $E^{*}$ and the equivalent radius $R^{*}$ are calculated as follows:

(4) $$\frac{1}{E^{*}}=\frac{1-{v_{1}}^{2}}{E_{1}}+\frac{1-{v_{2}}^{2}}{E_{2}}$$

(5) $$\frac{1}{R^{*}}=\frac{1}{R_{1}}+\frac{1}{R_{2}}$$

where $E_{1}$, $v_{1}$, $R_{1}$ and $E_{2}$, $v_{2}$, $R_{2}$ are the elastic modulus, Poisson’s ratio, and radius of particles 1 and 2, respectively.

**Figure 2:**
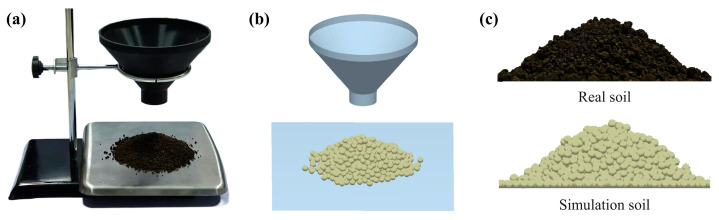
Funnel method to measure the angle of repose. (**a**) Experiment of the angle of repose test. (**b**) Simulation of the angle of repose test. (**c**) Angle of repose for real soil and simulated soil.

Some of the soil particle contact parameters were referenced from the literature and subsequently adjusted to optimize material and interaction parameters for accurate results, as summarized in Table 1 [12,30,35]. To ensure the reliability of the simulation, the contact parameters of soil particles from the three soil layers were calibrated using the funnel method [36], as illustrated in Figure 2. During calibration, soil samples were allowed to fall freely through a funnel, forming a stable stack. The accumulation profile was then captured from a perspective parallel to the stack. MATLAB was used to extract the repose angles from both sides of the soil stack in the images. Each experiment was repeated five times, and the average repose angle was recorded.

A corresponding simulation was constructed in EDEM using identical parameters to the physical experiment, and the tests were repeated in the virtual environment. A multiple regression analysis was performed on the experimental data to develop a repose angle regression model. After calibration, the error between the simulated and experimental repose angles was 3.40%, 4.18%, and 2.48% for the three soil types, respectively. These results indicate that the parameters listed in Table 1 were appropriate for the simulation.

**Figure 3:**
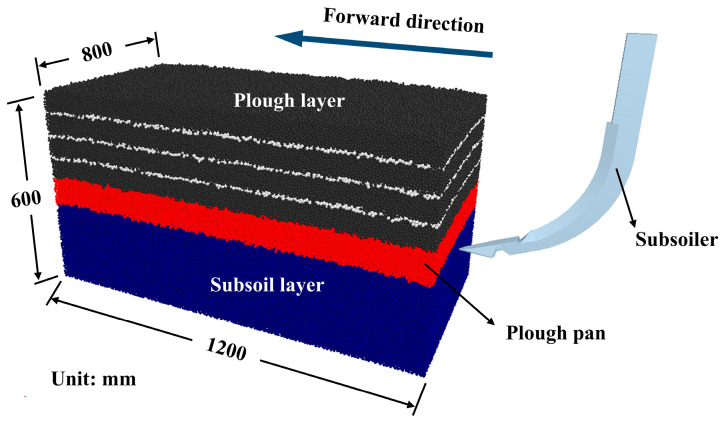
Simulation model of soil bin and subsoiler.

To ensure that the displacement of soil particles was not constrained by the soil bin boundaries or computational time, multiple trials were conducted, leading to the establishment of a soil bin with dimensions of 1200 mm × 800 mm × 600 mm. The soil bin was divided into three distinct soil layers to closely replicate actual field conditions. From top to bottom, these layers included the plough layer (0–260 mm, black), plough pan (260–350 mm, red), and subsoil layer (350–600 mm, blue). Additionally, white marker particles were embedded in the plough layer to track soil disturbance. The subsoiler model was then imported and aligned along the longitudinal axis of the soil bin, as illustrated in Figure 3.

#### 2.2.2. Working Parameters and Performance Evaluation Indexes of Subsoiler in DEM Analysis

In field conditions, the plough pan typically forms within a depth range of approximately 300 mm. Additionally, due to repeated machine operations, the upper subsoil layer at around 400 mm depth is also subject to compaction [37]. Based on these considerations, the working depth of the subsoiler in the simulation was set to 350 mm, which is a commonly selected depth for subsoiling operations in practice. The forward speed of the subsoiler significantly affects both the draft force and the soil disturbance area. In Northeast China, the typical subsoiling speed ranges from 3 to 7 km/h [14]. Therefore, three speed levels of 3, 5, and 7 km/h were selected to analyze the forces and soil disturbance characteristics of the subsoiler at different speeds in this study. To ensure computational accuracy, an appropriate time step was selected, considering factors such as model complexity and computing capacity. The Rayleigh time step calculated by the software was 6.82 × 10⁻^5^ s, and the fixed time step was set to 3.41 × 10⁻^6^ s, i.e., 5% of the Rayleigh time step. The total simulation time was 2 s, allowing the subsoiler to fully traverse the soil bin. The data saving interval was set to 0.01 s.

#### 2.2.3. Performance Evaluation Indexes of Subsoiler in DEM Analysis

**Figure 4:**
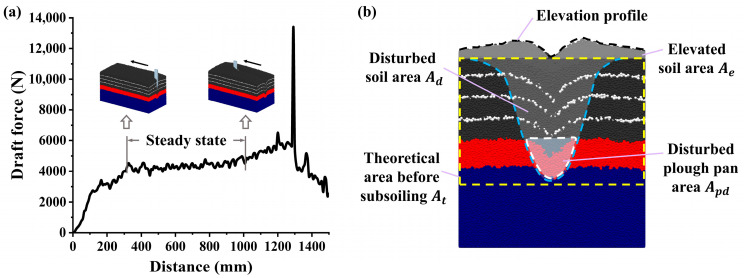
(**a**) Example of simulated draft force; (**b**) graph of soil disturbance after subsoiling in EDEM.

Draft force is a key indicator for evaluating subsoiler performance. The draft force gradually increased before stabilizing and fluctuating around a constant value as the subsoiler entered the soil bin. However, near the soil bin boundary, the resistance increased significantly due to boundary effects. Therefore, the draft force during the steady-state phase was selected for performance analysis, as shown in Figure 4a. The primary function of the subsoiler is to break the plough pan, facilitating the transport of water and nutrients to crop roots [38]. At the same time, it is essential to minimize the disturbance of the topsoil. Excessive disturbance in the surface soil layer can lead to soil erosion and negatively affect moisture retention and crop growth. Consequently, the extent of soil disturbance is an important index for assessing tillage quality.

In DEM simulations, the internal soil disturbance profile can be evaluated to determine the effects of the subsoiler. In this study, the disturbed soil regions after subsoiling were classified, as shown in Figure 4b, to assess the specific impact of the subsoiler. Particles in different regions were displayed separately, and their boundaries were marked. These contour curves were imported into CAD software for fitting, and computational tools were used to determine the area of the disturbed regions. The soil disturbance coefficient d, soil bulkiness q, and plough pan disturbance rate w were used to evaluate the soil disturbance effects of the subsoiler. These parameters were calculated as follows:

(6) $$d=\frac{A_{d}}{A_{t}}\times 100\%$$

(7) $$q=\frac{A_{e}}{A_{t}}\times 100\%$$

(8) $$w=\frac{A_{pd}}{A_{p}}\times 100\%$$

where $A_{t}$ refers to the cross-sectional area from the soil surface to the theoretical subsoiling bottom after subsoiling, as represented by the yellow dashed line in the figure; $A_{d}$ refers to the disturbed soil area, shown by the blue dashed line. $A_{e}$ represents the elevated soil area after the simulation of the subsoiling, indicated by the black dashed line in the figure. $A_{p}$ is the cross-sectional area of the plough pan when it is undisturbed. $A_{pd}$ represents the disturbed area below the plough pan, which is a part of the disturbed soil area.

In summary, the indicators for evaluating the tillage performance include the draft force of the subsoiler, soil disturbance coefficient, soil bulkiness, and the disturbance rate of the plough pan. These indicators will be analyzed in detail below.

### 2.3. Field Experiments

**Figure 5:**
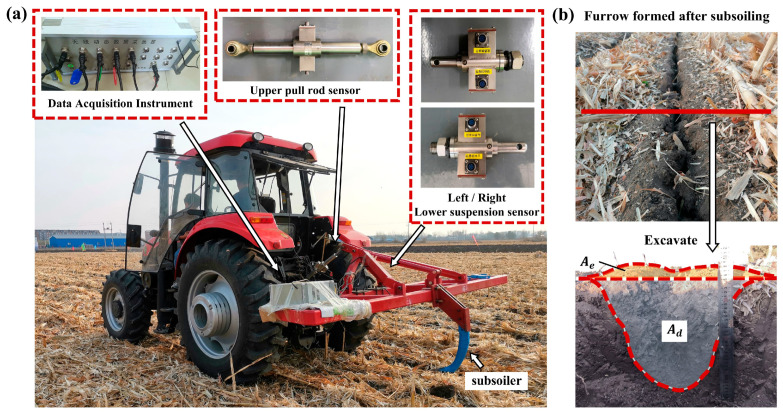
Field experiment. (**a**) The tractor and force measuring device in the field experiment. (**b**) Soil disturbance profile from a subsoiler.

Field experiments were conducted to validate the actual performance of the subsoiler. The designed subsoiler was tested in the field in Gongzhuling City, Changchun, Jilin Province, China. A tractor (LX1504, YTO GROUP CORPORATION, Luoyang, China) was used as traction power for the subsoiler. During the experiments, the tillage depth of the subsoiler was maintained at 350 mm, consistent with the simulation conditions. Three different forward speeds were considered. The tractor traveled a total distance of 80 m, with the first and last 10 m used for speed adjustments, while the middle 60 m served as the data acquisition region. Each test was repeated three times. To measure draft force, a B1-S sensor (China Academy of Aerospace Aerodynamics, Beijing, China) was selected in the experiment. The mounting frame was connected to the tractor via an upper pull rod sensor and lower suspension sensors. The horizontal draft force was determined as the vector sum of three horizontal components measured by the upper link and lower suspension sensors. The sensors operated at a sampling frequency of 5 Hz, and data were transmitted to a computer via Bluetooth. The tested subsoiler was securely attached to the mounting frame using bolts, as shown in Figure 5a. To evaluate the disturbed area created by the subsoiler during the field experiments, three random locations within the subsoiled furrow were selected for each test. At these locations, hollows were excavated, loose soil was removed, and cross-sectional images of the soil profile were captured. Due to the complexity of the field soil environment and the difficulty in distinguishing the interfaces between different soil layers, the soil surface and the disturbance contour were manually delineated in the images, as shown in Figure 5b. These profiles were then analyzed using AutoCAD 2018 software which was then used to calculate the elevated area $A_{e}$ and disturbed area $A_{d}$.

## 3. Results and Discussion

### 3.1. Draft Force of Subsoiler in DEM Simulation

**Figure 6:**
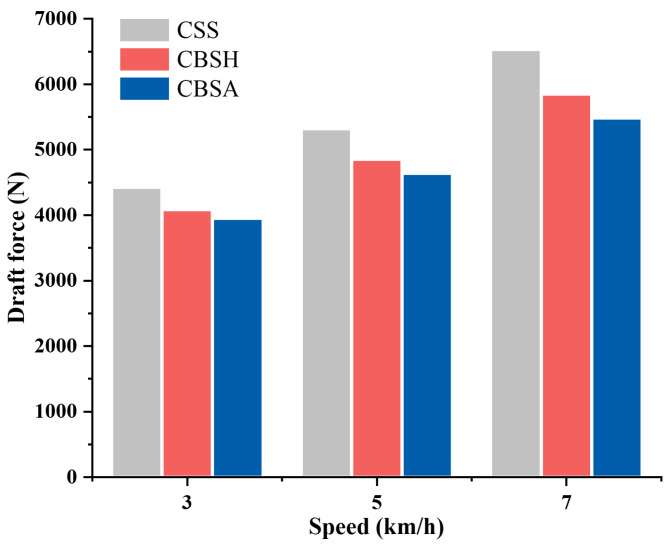
Draft force of subsoilers at different speeds in DEM simulation.

Figure 6 presents the draft force measurements of the three subsoiler types at different forward speeds in the simulation. As shown in the figure, both coupling bionic subsoilers (CBSH and CBSA) exhibited a draft force reduction in performance under different tillage speeds. Specifically, the draft force of CBSH was reduced by 7.70–10.43% compared to CSS, while that of CBSA was reduced by 10.65–16.02%**.** At each forward speed level, CBSA demonstrated the lowest draft force, the draft force at 3 km/h, 5 km/h, and 7 km/h was 3954 N, 4640 N, and 5485 N, respectively. The subsoiling speed had a significant impact on resistance. As the forward speed increased, all subsoilers showed a noticeable increase in draft force. This trend can be attributed to higher speeds which intensify the compression and shearing effects on the soil layer [39]. Notably, the draft reduction rate of the bionic subsoilers improved as the forward speed increased. At 7 km/h, the coupling bionic subsoilers showed the highest draft reduction rate. CBSH and CBSA reduced the draft force by 10.43% and 16.02%, respectively, compared to CSS.

**Figure 7:**
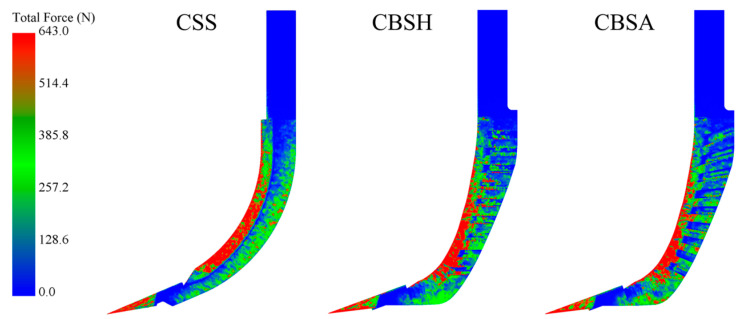
Visual force cloud diagram of three subsoilers at 5 km/h speed.

The force distribution on the subsoiler shank during the subsoiling operation can be visualized through force cloud diagrams. Figure 7 shows the visual force cloud diagrams of the three subsoilers at the speed of 5 km/h, where red represents regions experiencing higher forces, while blue represents regions subjected to lower forces. It was observed that, at the same speed, the red regions at the cutting edge of bionic subsoilers were significantly smaller than those of the conventional standard subsoiler. For the bionic subsoilers, high-stress regions were mainly concentrated in the lower portion of the cutting edge. This distribution reflects the effectiveness of the bionic curved edge design, which improves sliding and cutting performance, enhances stress dispersion, and reduces energy consumption and soil resistance during operation.

**Figure 8:**
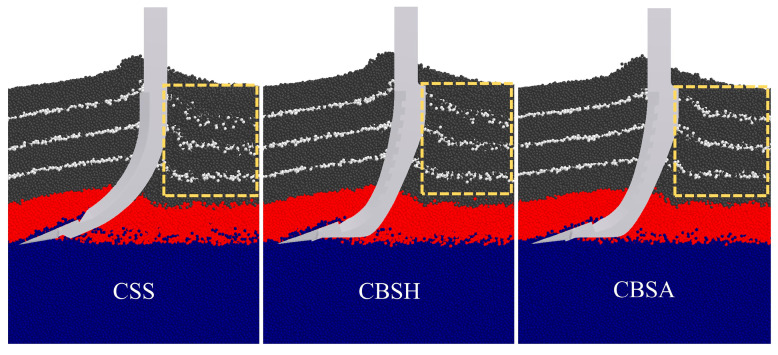
Cross-section of soil bin during forward movement of different subsoilers.

Examining the lateral surfaces of the subsoiler shank, it was found that the blue regions of the bionic subsoilers were larger and more widely distributed than those of the conventional subsoiler. This indicates that the force acting on the lateral surface of the bionic subsoilers was lower than that on the conventional standard subsoiler. This reduction in force is attributed to the presence of the bionic groove structure, which transforms soil particle movement from a disordered to an ordered state. To a certain degree, this trend alters the interaction between the subsoiler and the soil. Moreover, the discontinuous contact generated by the non-smooth structure helps to reduce friction and adhesion between the soil and the subsoiler. Among the three subsoilers, CBSA exhibited the smallest red and green regions on the force cloud diagram, which visually indicated the lowest resistance. White particles within the plough layer were used to observe the soil state during subsoiling. As shown in Figure 8, the white particles behind the CSS were more dispersed than those behind the coupling bionic subsoilers. Furthermore, CBSA had the most concentrated distribution of marker particles, and this phenomenon became more evident closer to the surface soil layer. This suggests that the groove structure with an angle provided superior guidance for soil particles, reducing soil disturbance during subsoiling. This soil movement effect likely contributed to CBSA achieving the lowest average draft force among all tested subsoilers.

### 3.2. Soil Disturbance of Subsoiler in DEM Simulation

**Figure 9:**
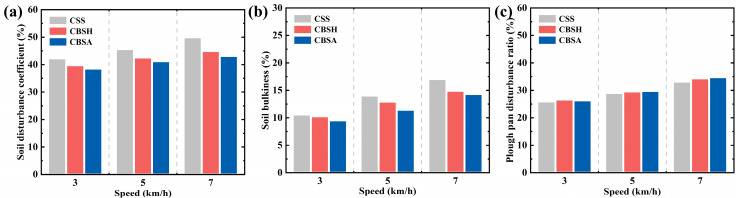
The values of soil disturbance characteristics at different speed ranges. (**a**) Soil disturbance coefficient. (**b**) Soil bulkiness. (**c**) Plough pan disturbance coefficient.

To evaluate the soil disturbance characteristics of the subsoilers, the soil disturbance parameters of the three subsoilers at varying speeds were calculated, as illustrated in Figure 9. An increase in operating speed resulted in higher values of the soil disturbance coefficient, soil bulkiness, and plough pan disturbance rate. This trend is consistent with previous studies, indicating that higher tillage speeds result in more aggressive disruption of the soil [40,41].

When the working speed increased from 3 km/h to 7 km/h, the disturbance area caused by the standard subsoiler was consistently larger than that of the coupling bionic subsoilers, as shown in Figure 9a. Compared to CSS, CBSA reduced soil disturbance by 8.94–13.57% across different speeds, while CBSH exhibited a reduction of 5.91–9.90%. At the same speed, CBSA resulted in the least soil disturbance, which also correlated with a lower draft force. This finding is consistent with the draft reduction effect observed in the coupling bionic subsoiler. Furthermore, these results suggest that the angle of the bionic ribbed structure on the subsoiler influences soil disturbance characteristics, which is similar to the results in the literature [14].

Minimizing soil bulkiness is desirable, as excessive topsoil disruption can promote moisture evaporation and reduce soil water retention capacity. As shown in Figure 9b, at different operating speeds, the soil bulkiness of the coupling bionic subsoilers was lower than that of the standard subsoiler. Specifically, CBSH reduced soil bulkiness by 2.84–12.63%, while CBSA achieved a reduction of 9.84–18.41%, effectively decreasing the exposure of surface soil. Notably, CBSA exhibited significantly lower soil bulkiness than CBSH at the same working speeds, indicating that the angled groove structure more effectively limits surface disturbance.

A higher plough pan disturbance rate is generally associated with improved subsoiling effectiveness. As shown in Figure 9c, the disturbance rate of the plough pan increased with speed. At 7 km/h, CBSA achieved the highest plough pan fragmentation rate of 34.77%, which was 4.7% higher than that of the CSS. At lower speeds, the plough pan disturbance rates of the coupling bionic subsoilers and the standard subsoiler were relatively similar. This result may be attributed to the fact that the shape of the subsoiler tine greatly influences the degree of disturbance in the plough pan. Since all the subsoilers used in this study were equipped with standard sweep tine, the disturbance rates in the plough pan caused by the three types of subsoilers were relatively similar at the same operating speed.

These simulation results show that modifications to the subsoiler structure influence soil disturbance characteristics and dynamic soil behavior. The coupling bionic subsoilers exhibited a superior disturbance performance in simulations. Among the tested designs, CBSA achieved the most favorable disturbance effect, characterized by lower surface soil bulkiness and a reduced soil disturbance rate, which are beneficial for crop root development.

### 3.3. Field Experiment Results and Analysis

**Figure 10:**
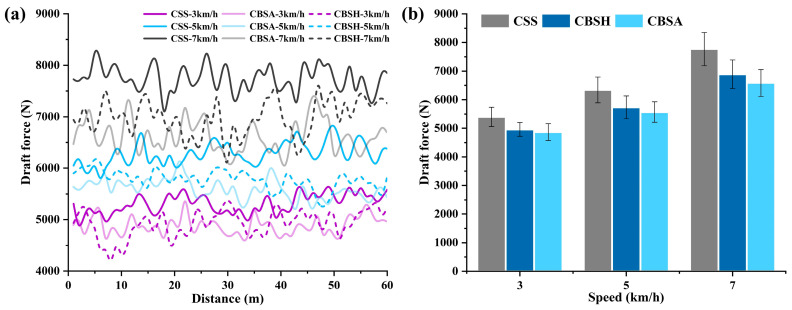
Field experiment results. (**a**) The draft force of subsoilers during operation. (**b**) Average draft force of the subsoilers in field experiment.

Figure 10a illustrates the variation in draft force with distance for the three subsoilers at different speeds in the field experiments. The draft force exhibited a fluctuating pattern, primarily due to the cutting and sliding interactions between the subsoiler and the soil. Specifically, the crescent soil deformation area formed ahead of the subsoiler underwent cyclic failure within the soil layer [42,43]. The field experiment data curves showed that both CBSH and CBSA demonstrated draft reduction effects across different speeds, suggesting that the incorporation of bionic structures provided the subsoiler with improved draft reduction characteristics.

The average draft force measured for the three subsoilers under different speeds is shown in Figure 10b. The average draft force of coupling bionic subsoilers was reduced by 11.06%. At the working speed of 7 km/h, the highest reduction in draft force was achieved. The average draft forces for CBSA and CBSH were 6587 N and 6891 N, respectively, representing reductions of 15.23% and 11.31% compared to the draft force of 7770 N for the conventional standard subsoiler. This improvement can likely be attributed to the design considerations that differentiated the working conditions of the cutting edge and the lateral surface of the shank. The bionic structure of the cutting edge is more conducive to cutting the soil and breaking the soil wedge in front of the blade in advance, thereby reducing the resistance. Meanwhile, the bionic structure on the subsoiler side edge transformed disordered soil movement into an organized flow, preventing excessive soil particle collision and interference. In other words, the bionic structure provided directional guidance for fractured soil, reducing the contact area and further decreasing the draft force.

Working speed had a positive effect on the draft reduction rate of the coupling bionic subsoilers. As the speed increased from 3 km/h to 5 km/h and then to 7 km/h, the growth rates in the draft force for CBSA were 14.41% and 18.29%, while for CBSH they were 15.60% and 20.21%, respectively. These values were all lower than the corresponding increases observed for CSS, which were 17.41% and 22.56%. These results suggest that the bionic structural design mitigated the increased impact of speed on tillage resistance. At the same speed, CBSA exhibited a lower draft force than CBSH, and its draft force growth rate was also lower, indicating that CBSA provided a superior draft reduction performance compared to CBSH. At higher speeds, the draft force of the subsoilers exhibited greater fluctuations. These fluctuations can be attributed to two primary factors—the increase in soil shear strength and frictional resistance with speed, as well as variations in field soil properties—both of which contributed to the observed resistance fluctuations.

The draft force measured in field experiments was higher than in the simulations, with a maximum error of less than 20%. Although the draft force was higher in the field experiment, the trend of the change in the draft force was the same. This difference is typically due to the complexity of actual environmental field conditions. The presence of plant roots and crop residue in the field is unavoidable and generally increases the draft force during tillage operation [44]. Therefore, the simulation results are considered reasonable and acceptable. The soil simulation model can be effectively used to analyze and verify the mechanical characteristics during subsoiling. It is worth noting that both simulation and field experiments demonstrated an increase in the draft reduction rate of coupling bionic subsoilers with increasing speed. Therefore, under the premise of ensuring operational quality, increasing the working speed of the bionic subsoilers can improve efficiency while saving more energy compared to low-speed operation.

The soil disturbance areas of the subsoilers at different speeds are presented in Table 2. Significant variations in disturbance were observed among the three subsoilers across different working speeds. Both Ad and Ae increased with increasing working speed. The trends in Ad and Ae for the three subsoiler types were generally consistent with the simulation results, indicating that the soil model established in the DEM simulation can be effectively used to analyze and validate the subsoiling effects in the specific soil conditions examined in this study. As shown in Table 2, the smallest disturbance area was observed at 3 km/h, while the largest occurred at 7 km/h. This may be attributed to the increasing volume of the soil wedge formed in front of the subsoiler as speed increases, thereby enlarging the disturbed soil area. Additionally, with the increase in working speed, the disturbed soil obtains a higher velocity, exerting greater pressure on the adjacent soil and further expanding the total disturbance area. This finding is consistent with the effect of the tillage component speed on soil disturbance reported in the literature [45,46]. Furthermore, a higher speed will also lead to an increased disturbance of the soil on both lateral sides of the subsoiler shank [47]. Compared with the disturbance results of the subsoiler at the same speed, the disturbance area caused by the standard subsoiler is larger than that caused by the coupling bionic subsoiler. This shows that the bionic structure effectively changes the movement state of these soils and reduces the soil disturbance area. Therefore, the bionic structure is highly important to reduce the total disturbance of soil. Among the subsoilers, CBSA consistently resulted in the smallest disturbed area, making it a preferable option for subsoiling operations.

The coupling bionic subsoilers exhibited reduced surface disturbance compared to the standard subsoiler. Compared to the CSS, the evaluated soil area of CBSA decreased by 9.68% at 3 km/h, 12.65% at 5 km/h, and 13.96% at 7 km/h. For CBSH, the reductions were 7.87%, 9.87%, and 11.67%, respectively. These results demonstrate the superior capacity of the coupling bionic subsoilers to minimize surface soil disturbance, thereby enhancing the soil backfill rate. Among them, CBSA exhibited the lowest Ae value. Excessive disturbance of surface soil can lead to increased exposure, promoting moisture evaporation and loss. The CBSA design resulted in improved surface soil conditions, producing a smoother ridge–furrow interface, which helps to improve soil moisture content [48].

In summary, the soil model established in the DEM effectively supports the analysis and validation of subsoiling effects. Compared with the standard subsoiler, the coupling bionic subsoilers not only reduced draft force but also improved soil disturbance efficiency, better meeting the agronomic requirements of draft reduction and low energy consumption. These advantages contribute to improved crop root development and increased yield. In addition to enhancing the performance of the subsoiler, the successful application of this coupling strategy provides a novel approach for the development of soil-engaging components, offering more effective solutions to complex engineering challenges. For example, in fields such as construction and transportation where soil penetration and excavation are required, reducing resistance and soil adhesion is critical for improving operational efficiency. This bionic coupling design method demonstrates broad application potential in these fields.

## 4. Conclusions

In this study, two types of coupling bionic subsoilers were designed using the claw toes of the badger and the scales of the pangolin as bionic models. A layered soil model was established based on actual soil conditions, and the resistance and disturbance characteristics of bionic subsoiling were successfully simulated using DEM. The simulation analysis demonstrated that the coupling bionic subsoilers exhibited low resistance characteristics at low, medium, and high working speeds. Compared with CSS, the draft force of CBSH was reduced by 7.7–10.43%, while that of CBSA was reduced by 10.65–16.02%, indicating that CBSA achieved a better drag reduction performance. In addition, the coupling bionic subsoiler effectively reduced surface soil bulkiness and soil disturbance rates, achieving improved subsoiling performance. Furthermore, the performance of the coupling bionic subsoiler was verified through field experiments. In the field tests, the coupling bionic subsoilers demonstrated an excellent draft reduction performance, with an average reduction of 11.05% compared to the standard subsoiler. At a working speed of 7 km/h, CBSA achieved the best draft reduction effect, with a reduction of 15.23%. These findings contribute to the development of more efficient subsoilers by reducing draft force and optimizing soil disturbance, making them more in accordance with conservation tillage practices.

## Figures and Tables

**Table 1:** Materials and interaction parameters used in EDEM.

Parameters	Plough Layer	Plough Pan	Subsoil Layer
Soil particle radius (mm)	4	4	4
Soil density (kg/m^3^)	2580	2630	2610
Poisson’s ratio of soil	0.30	0.33	0.31
Shear modulus of soil (Pa)	1 × 10^8^	1.02 × 10^8^	1.05 × 10^8^
Soil particle model surface energy (J/m^2^)	2.79	4.52	3.25
Number of soil particle models	405,723	138,335	418,013
Coefficient of restitution, soil–soil	0.65	0.62	0.68
Coefficient of static friction, soil–soil	0.50	0.51	0.52
Coefficient of rolling friction, soil–soil	0.07	0.11	0.12
Density of steel (kg/m^3^)	7850	7850	7850
Poisson’s ratio of steel	0.30	0.30	0.30
Shear modulus of steel (Pa)	7.9 × 10^10^	7.9 × 10^10^	7.9 × 10^10^
Coefficient of restitution, soil–steel	0.29	0.26	0.27
Coefficient of static friction, soil–steel	0.57	0.49	0.50
Coefficient of rolling friction, soil–steel	0.3	0.28	0.27

**Table 2:** Comparison of average disturbed soil area of subsoiler at different speeds.

Parameters	Speed	CSS	CBSH	CBSA
$A_{d}$ (mm^2^)	3 km/h	72,930 a	69,583 b	69,809 c
	5 km/h	79,953 a	75,309 b	75,973 c
	7 km/h	83,199 a	78,600 b	76,706 c
$A_{e}$ (mm^2^)	3 km/h	12,976 a	11,955 b	11,720 b
	5 km/h	13,836 a	12,470 b	12,086 c
	7 km/h	14,907 a	13,167 b	12,470 c

Note: Statistical differences *p* < 0.05. a, b, and c indicate whether the difference between values is significant. If the letters following the same variable are the same, the differences are not significant; if the letters are not the same, the differences are significant.

## Data Availability

The data presented in this study are available on request from the corresponding author.
